# Dataset for detecting motorcyclists in pedestrian areas

**DOI:** 10.1016/j.dib.2023.109610

**Published:** 2023-09-22

**Authors:** Nicolás Hernández Díaz, Yersica C. Peñaloza, Y. Yuliana Rios, Juan Carlos Martinez-Santos, Edwin Puertas

**Affiliations:** a Computer Eng., Universidad Tecnologica de Bolívar, Km 1 vía a Turbaco, Cartagena, 130010, Bolívar, Colombia; b Electronic Eng., Universidad de Pamplona, Km 1 vía Bucaramanga, Pamplona, 543050, Norte de Santander, Colombia; c Mechanic Eng., Universidad Industrial de Santander, Calle 9, Carrera 27, Bucaramanga, 610101, Santander, Colombia

**Keywords:** IoT, Raspberry Pi, CCTV, Artificial vision, Data set construction, Multiclass object classification models

## Abstract

This paper presents a semi-automated, scalable, and homologous methodology towards IoT implemented in Python for extracting and integrating images in pedestrian and motorcyclist areas on the road for constructing a multiclass object classifier. It consists of two stages. The first stage deals with creating a non-debugged data set by acquiring images related to the semantic context previously mentioned, using an embedded device connected 24/7 via Wi-Fi to a free and public CCTV service in Medellin, Colombia. Through artificial vision techniques, and automatically performs a comparative chronological analysis to download the images observed by 80 cameras that report data asynchronously. The second stage proposes two algorithms focused on debugging the previously obtained data set. The first one facilitates the user in labeling the data set not debugged through Regions of Interest (ROI) and hotkeys. It decomposes the information in the nth image of the data set in the same dictionary to store it in a binary Pickle file. The second one is nothing more than an observer of the classification performed by the user through the first algorithm to allow the user to verify if the information contained in the Pickle file built is correct.

Specifications Table

**Table:** 

Subject	Data Engineering
Specific subject area	The presented Dataset involves classes such as pedestrians, motorcyclists, and their respective interactions with the road themselves through filters (labels in YOLO txt format) applied to the raw images; this structure pretends to give to the research community a possible Dataset to train IA models for **Intelligent traffic light systems**.
Data format	1. Raw 2. Filtered Raw, Analyzed, Filtered
Type of data	• Image • YOLOs txt Labels Table, Image, Chart, Graph, Figure
Data collection	The raw Dataset was acquired using an embedded device (Raspberry Pi W2) connected 24/7 via WiFi to a free and public CCTV service in Medellin, Colombia, over the internet, and the raw Dataset was filtered a debugged into YOLO text annotations using the same device and the tools made and report in this article.
Data source location	Free and public CCTV service operated by the Ministry of Mobility of Medellín in Medellin, Colombia.
Data accessibility	Repository name: https://zenodo.org/ Data identification number: 10.5281/zenodo.7935299 Direct URL to data: https://zenodo.org/record/7935299 Instructions for accessing these data: Not applicable

## 1 Value of the Data

• One of the great difficulties the scientific community faces when developing artificial intelligence models is obtaining quality, structured, and publicly accessible data, especially if the semantic context involves classes such as pedestrians, motorcyclists, and their respective interactions with the road as themselves. In addition, constructing a data set is a task that requires a lot of time and order, even more so if there are no tools or methodologies that facilitate their generation and development.
• Anyone looking for labeled data where the semantic context of his project is near to the concept of Intelligent traffic light systems assisted by artificial vision.
• It is possible to generalize that the state of the art is searching for more optimal and efficient techniques for constructing robust data sets that do not require human intervention as much as possible. However, their reproducibility and comprehension have become more complex, causing a knowledge gap in users looking for less complicated and more uncomplicated techniques to apply to construct depurate image data.
• So, this work proposes a specific and straightforward methodology to get the same data into the same semantic context giving his reader tools and steps to get a labeled Dataset.
• As a reference of how the scientific community can apply this type of data to their projects, please check the work developed in [1] which represents just an example of the value and the usefulness of the Dataset generated.

## 2 Data Description

**Fig. 1:**
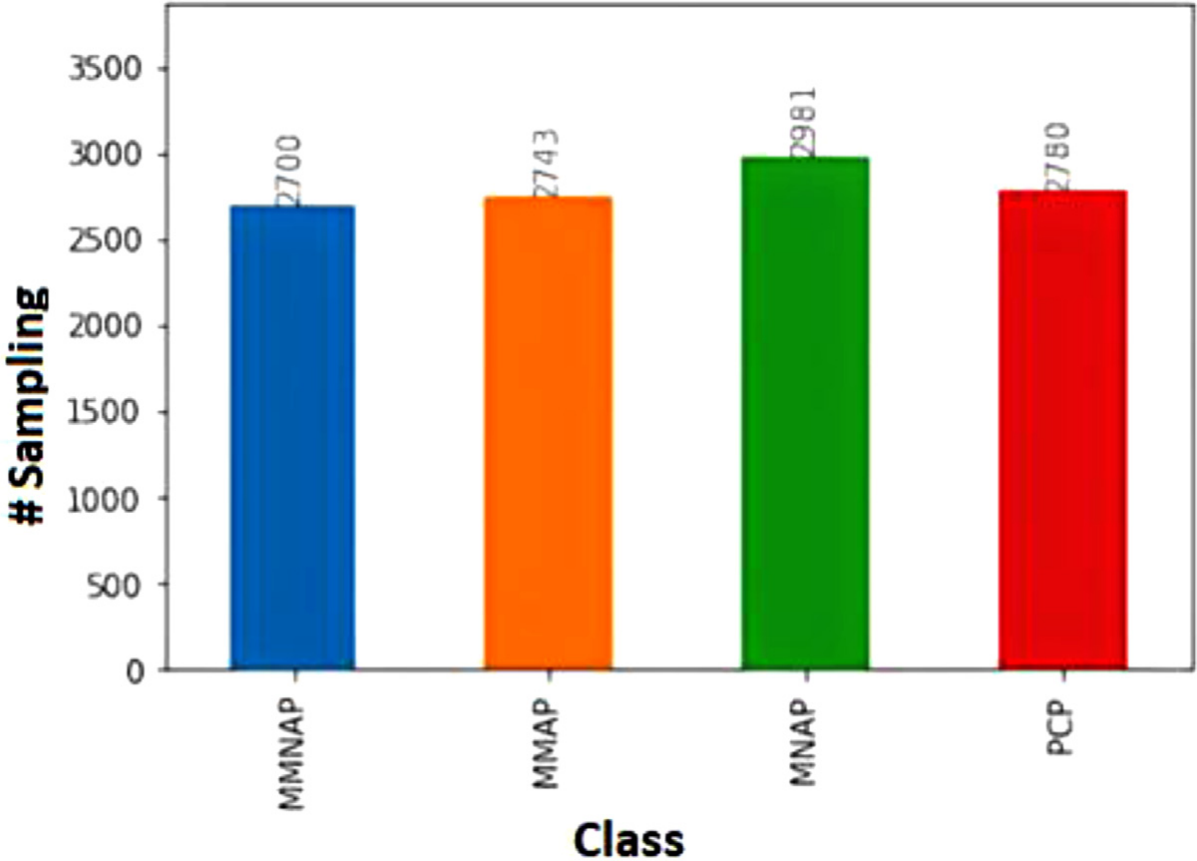
Dataset's Distribution.

The file PedestrianDataSet.zip contains two directories and one file, the directory named “**images**” contains all the images captured; the directory named “**etiquetas**” contains all annotations done based on four classes ordered as [MMNAP, MMAP, MNAP, PCP] in YOLO txt format, the name's correspondence with the label is [motorcycle with motorcyclist not in a pedestrian area, motorcycle with a motorcyclist in a pedestrian area, motorcycle without motorcyclist in a pedestrian area, pedestrian in crosswalk]; the file Distribution.png is an image that explains how the Dataset is distributed and it is illustrated in Fig. 1.

The classes chosen were selected pursuing a possible development of a system that allows to discriminate between a pedestrian who is causing the regular traffic flow doesn't work and a motorcycle driver who is occupying the pedestrian cross and doesn't allow to the pedestrians cross the street.

The dataset contains 6324 images in .jpg format for a total of 643.9 MB, the resolution of each image is 1280 × 720 (HD), and the weight per image is under 120 KB. The MMNAP label has 2700 samples, the MMAP label has 2743 samples, the MNAP label has 2981 samples, and the PCP label has 2780 samples. We obtained the images from a closed-circuit television CCTV free public access located in Medellín, Colombia, consisting of 80 cameras on the road. These images were captured mainly during daylight hours. The format of the name of each image is “image (camera number) _(date format"AAAAMMDD”)_(time format “hhmmss”), where (AAAA) is the year, (MM) is the month, (DD) is the day, (HH) is the 24- hour, (MM) is the minute and (SS) is the second, each YOLO txt annotation follows the same formating.

## 3 Experimental Design, Materials and Methods

The methodology developed for constructing the Dataset consists of two main lines of execution. However, only the line of image acquisition from the CCTV server uses a Comparative Chronological Analysis to determine whether it is necessary to download the image observed by the nth camera at an instant t of time. The second line consists of the labeling process using rectangular-type ROIs and hotkeys together with the verification process of the labels performed.

### 3.1 Comparative chronological analysis for image download

Comparative Chronological Analysis is the inference process of analyzing the current image observed by the nth camera and the magnitude of the observable difference on a previous image from the same camera. The following equation represents the operation done on it to the data observed by each camera.

$$ACC\left(A,B\right)=\frac{1}{n}\sum_{i=1}^{n}\sum_{j=1}^{n}{\left(A_{\left(i,j\right)}-B_{\left(i,j\right)}\right)}_{\left(i,j\right)}$$

Where:

• **ACC (A, B)** Chronological Comparative Analysis returns a scalar value.
• **n** is **rows x columns** the number of rows times the number of matrix columns.
• **(i,j)** the state of indexing over the image's array
• **i** represents the actual row and **j** the actual column in the image's array.
• **A** the current image observed by the nth camera.
• **B** the previously stored image of the nth camera.
• **A(i, j)** the scalar value of the pixel registered in the position **(i,j)** of **A**.
• **B(i, j)** the scalar value of the pixel registered in the position **(i,j)** of **B**.

The criteria for downloading an image for the nth camera analysis depends on the value **ACC (A, B)** previously obtained, therefore,

$$BD=\left\{ \begin{array}{cc} True & \text{if}ACC(A,B)>threshold\\ False & \text{if}ACC(A,B)\leq threshold \end{array}\right.$$

Where:

• **BD** Flag to download.
• **ACC (A, B)** Chronological Comparative analysis (Scalar Value)
• **threshold** that sets the download criteria.

The Comparative Chronological Analysis for the Image Download method can ensure that the system does not store identical images based on the download criteria due to possible communication errors or the asynchronous update behavior the connected IP cameras typically connect to the CCTV. It achieves the construction of a raw data set that does not contain repeated or too similar images (threshold).

### 3.2 Region of interest (ROI) and hotkeys

After obtaining the raw data set, labeling based on the classes defined in the project is necessary. In an image, we represent the classes with two-dimensional data. Therefore, the user can extract the information of interest. A technique widely used by different manual labelers is using rectangular ROIs.

**Fig. 2:**
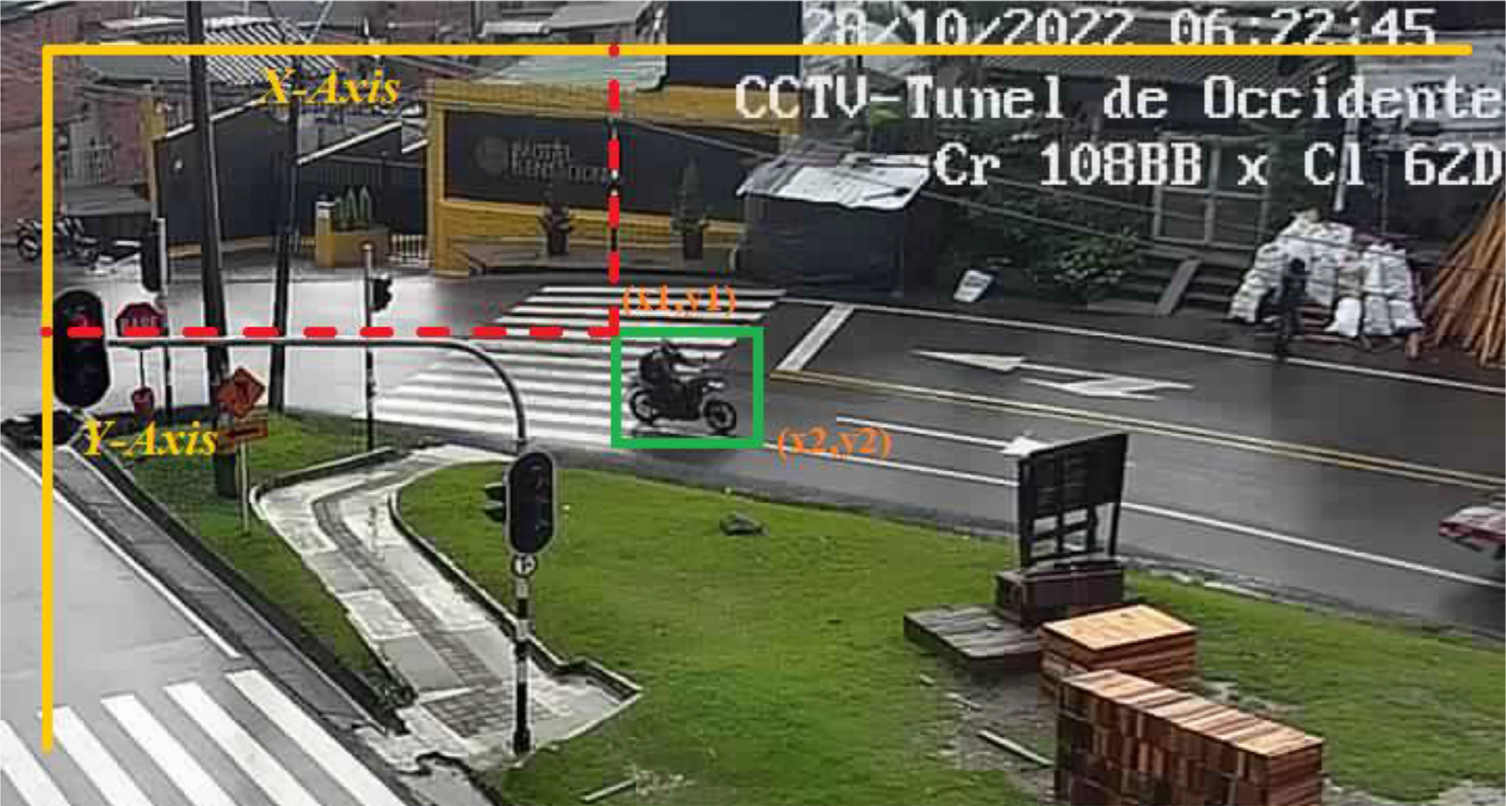
Example of a selected ROI for an object of a specific class.

***Rectangular ROIs*** is a tool that makes it possible to manually select a range of interest in an image by selecting the area in the image and returning a vector containing the indexing of the top starting vertex of the rectangle and the bottom opening vertex. Fig. 2 presents a sample of the data set built into this document, along with the ROI selection.

The fast access keys are a procedure based on the continuous sensing of the state of a key during the observation of the nth image or general sample so that each key is assigned a class or task. For this work, the following equation summarizes the cases.

$$Assign=\left\{ \begin{array}{cc} MMNAP & \text{if}keyState==A\\ MMAP & \text{if}keyState==B\\ MNAP & \text{if}keyState==C\\ PCP & \text{if}keyState==D\\ continueNext\text{Im}age & \text{if}keyState==Q \end{array}\right.$$

Were,

• Assign corresponds to the path within a dictionary where the cut-out, the ROI coordinates, and the class label should be stored.
• [MMNAP, MMAP, MNAP, PCP] are the distant classes recognized during the project.
• continue tells the system that the general sample information does not contain classes or that there are no more classes to classify.

**Fig. 3:**
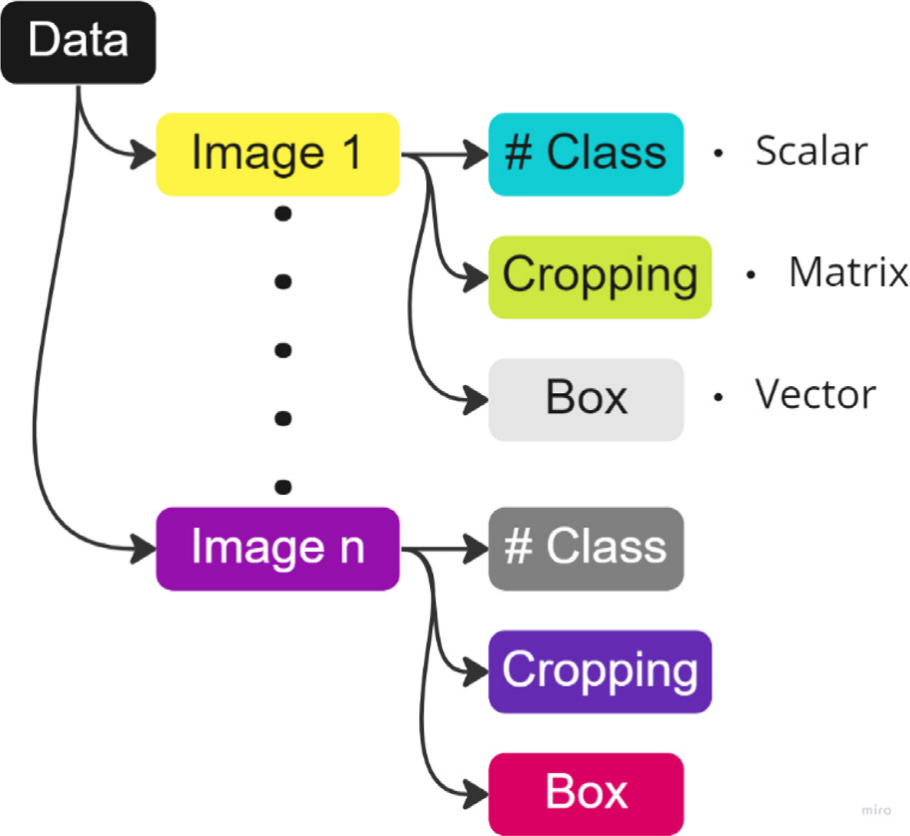
Structure of the obtained clean data set.

Combining both concepts makes building a refined and structured data set possible, as shown in Fig. 3.

### 3.3 Data source

The data come from a free CCTV comprising 80 cameras arranged publicly in Medellín [2]. They are part of the Intelligent Mobility System of Medellin (SIMM), a pioneering program in Colombia of the Medellín Mobility Secretary. It emerged in 2020 as a response to optimizing the city's road network.

The nature of the data represents a challenge to its excellent acquisition because although they are entirely free, each camera updates the information on the server after approximately five minutes and asynchronously for the other cameras.

### 3.4 Methodology

**Fig. 4:**
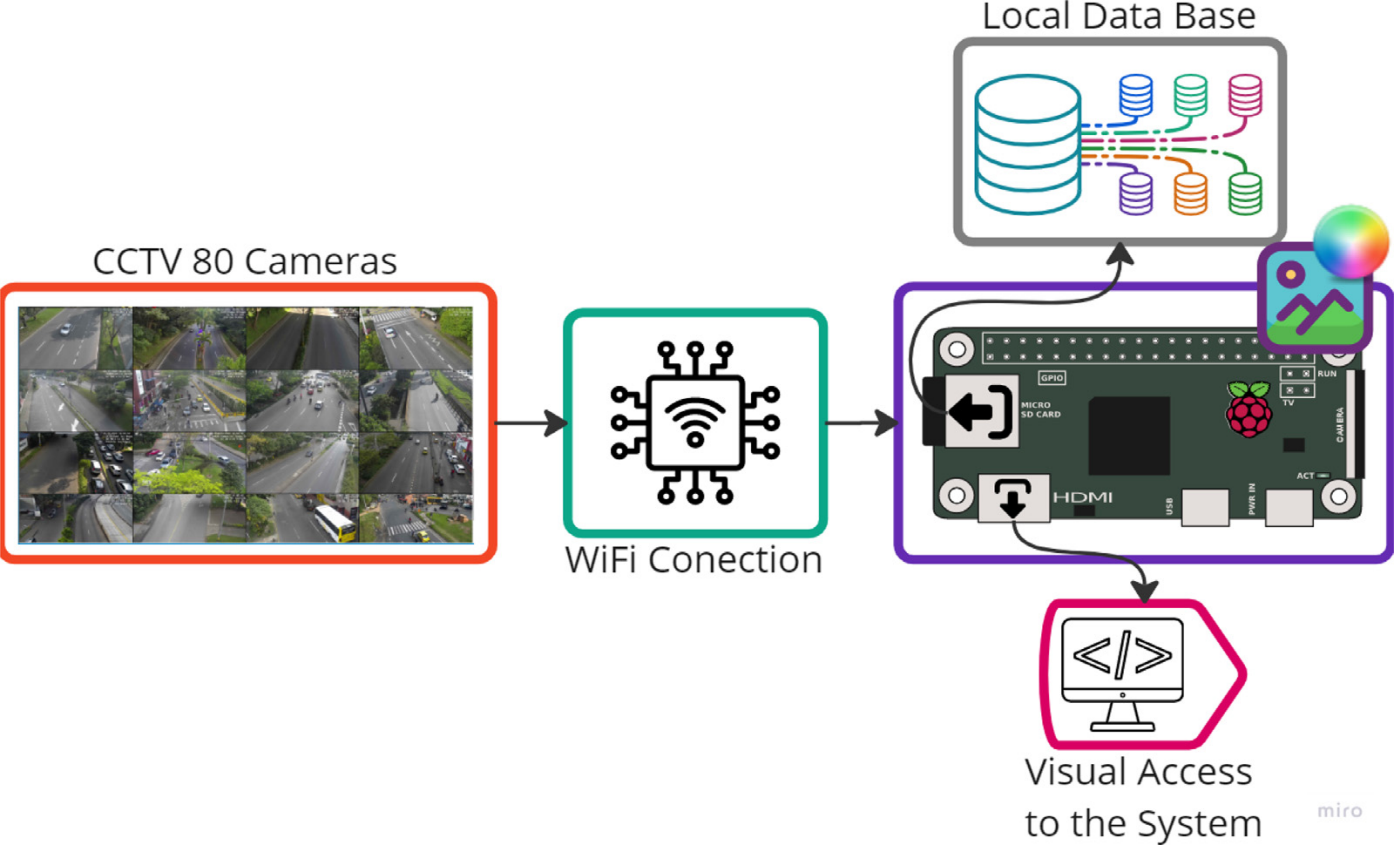
Methodology implemented.

Fig. 4 shows a diagram of the proposed methodology. This methodology adopts characteristics from works like [3], [4], [5], [6], [7]. As we can discern, the system depends on the availability and state of the data present in the CCTV. The request for information (RGB Image) is made to each camera independently through a Raspberry Pi Zero W with an internet connection.

This embedded device, employing a Comparative Chronological Analysis, downloads images in .jpg format to construct the raw data set during the time indicated by the user or until the execution is interrupted.

Finally, since the data stored by the system doesn't have annotations is necessary for the user to manually initialize the algorithm based on the selection of Regions of Interest (ROI) and hotkeys to decompose the information in the nth image of the raw data set into the required classes, thus getting the data set clean and ready to train an object classifier.

Therefore, it is convenient to indicate that the protocol to annotate each image depends on the user's abilities and comprehension of the tool to get the final data set.

## 4 Results

As a result, we developed four algorithms, which are:

• Image acquisition algorithm for construction of the raw data set.
• Algorithm for the process of labeling and building the clean data set.
• Algorithm for graphically reviewing or verifying the status of the debugged data set.
• Algorithm to make the conversion between pickle format design and YOLO.

### 4.1 Image acquisition algorithm for construction of the raw data set

Algorithm 1 illustrates the data acquisition process; this is according to the presented methodology and corresponds to the implementation of the Comparative Chronological Analysis For Image Download, the *flag* type variables are used to verify the internet connection and the CCTV server, the *old* and *new_check*variables correspond to the (A,B) variables respectively, which are used to determine how different is the B image concerning the B image.

**Algorithm 1:** Algorithm for Get the Data from URL.

Algorithm 1 is developed under the following sequential process,

✓ Scroll through the total number of cameras available in the CCTV server and assign to a variable the current observable information for the nth camera.
✓ If the data of the nth camera are available in the CCTV server and there is no previous image of the nth image in a local folder, the image is stored in .*jpg* format.
✓ If there is a previous image captured from the nth camera, the Comparative Chronological Analysis technique is applied to determine if the currently available image is different from the nth-1 image that was previously stored, to make the discrimination according to a threshold previously defined empirically.

**Fig. 5:**
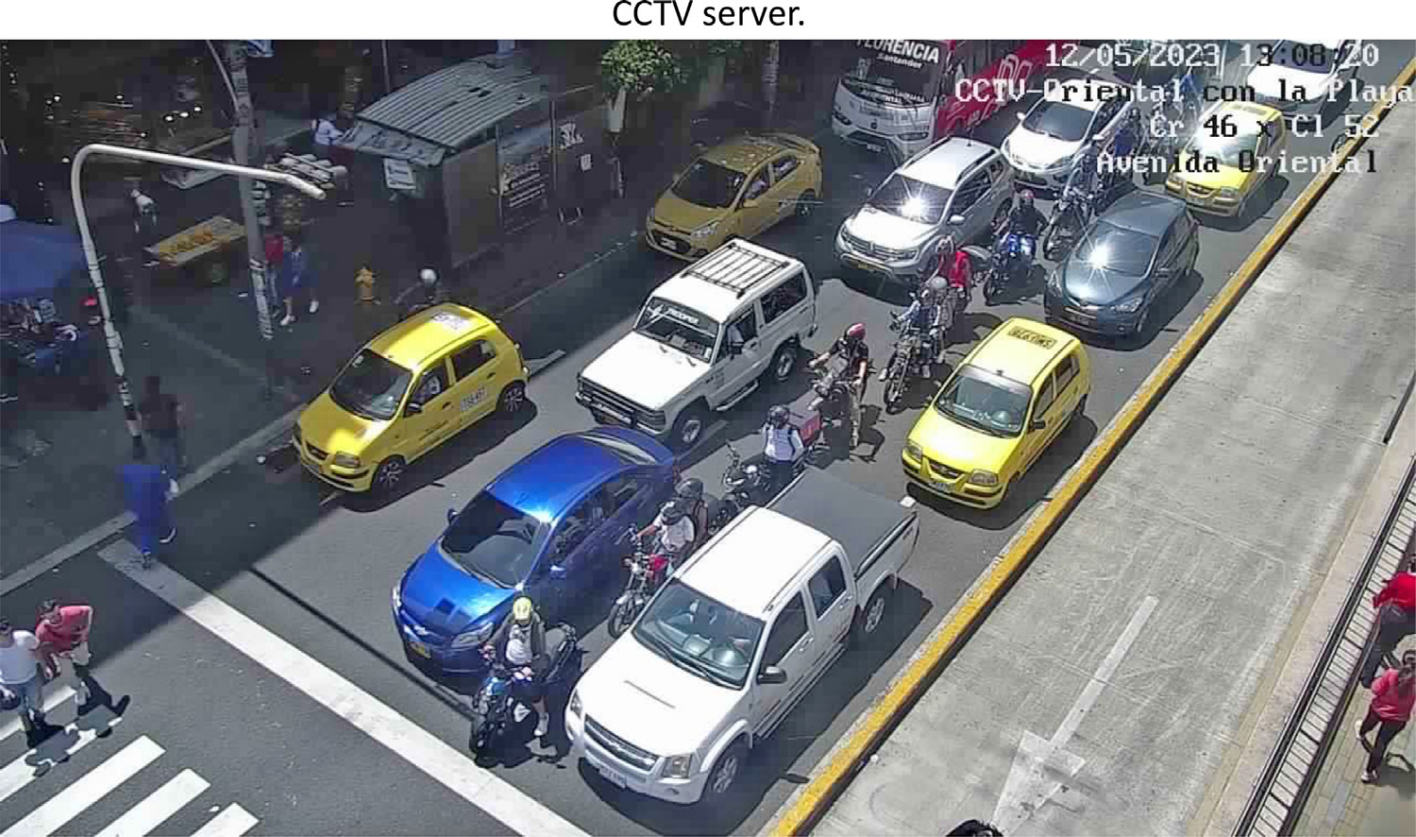
Example of the type of images acquired.

The Fig. 5 illustrates the type of images acquired by the acquisition system implemented; it is worth noting that as can be seen there is no control over the lighting, much less the angle of incidence at which the camera captures, i.e. the system is at the mercy of the data available on the CCTV server.

### 4.2 Algorithm for the process of labeling and building the clean or filtered data set

The code below shows how extracting Regions of Interest (ROI) was implemented and using hotkeys to direct the clipping, class, and bounding box object to the *Data* variable and the hierarchy in its data structure.

Algorithm 2 is developed under the following sequential process,

✓ Read and display to the user the nth image previously captured and stored in *.jpg* format.
✓ Activate the ROI-based selection environment which allows to define the bounding box of the nth object of interest within the nth image.
✓ Wait for the user to select one of the 4 hotkeys; each hotkey allows to relate the nth bounding box to one of the 4 classes of interest.
✓ Store the coordinates of the nth bounding box *box*, the snippet as image segment describing the bounding box *snippets* and the class index *class* in the range of [0–3] within a dictionary where the main key is the image name.

**Algorithm 2:** Algorithm for the labeling process and construction of the data set.

**Fig. 6:**
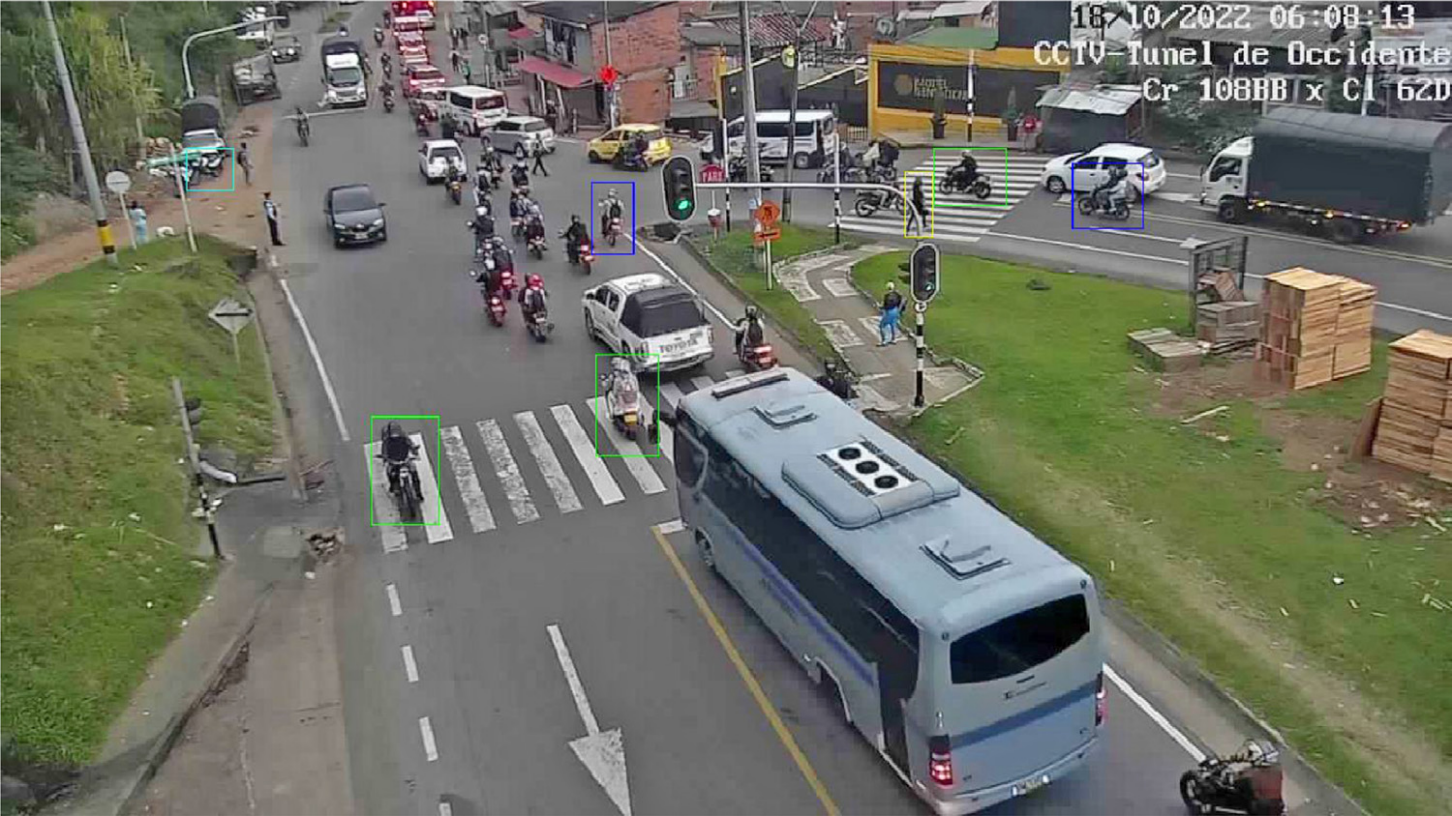
General operation of the labeling system.

Store the created dictionary in a .*pkl* file after finishing reading all previously captured and stored images in .*jpg* format. Similarly, at the end of the code, it can see the redefinition of the data structure by going from general samples in .jpg format to a single binary file in Pickle format that contains all the information necessary to train and validate a multiclass object classifier model. Fig. 6 shows the general use of this section.

### 4.3 Algorithm for graphically reviewing or verifying the status of the debugged data set

After completing the labeling process, using the code illustrated below, it is possible to review the labels made following the data hierarchy, presenting both the color assigned to the label and the object bounding box and the text of the class type.

Algorithm 3 is developed under the following sequential process,

✓ Read and display to the user the umpteenth image previously captured and stored in .*jpg* format.
✓ Read the .*pkl* file previously created and reassign its content to a dictionary type variable.
✓ Draw on the nth image read the content related to the name of the image on the dictionary, presenting the user with the object delimiter box of the nth object in the image together with the name of the class that was assigned.

**Algorithm 3:** Algorithm for reviewing or verifying the status of the debugged data set.

Wait the user wants to continue reading the remaining images until each and every image previously captured and stored in *.jpg* format is finished reading.

**Fig. 7:**
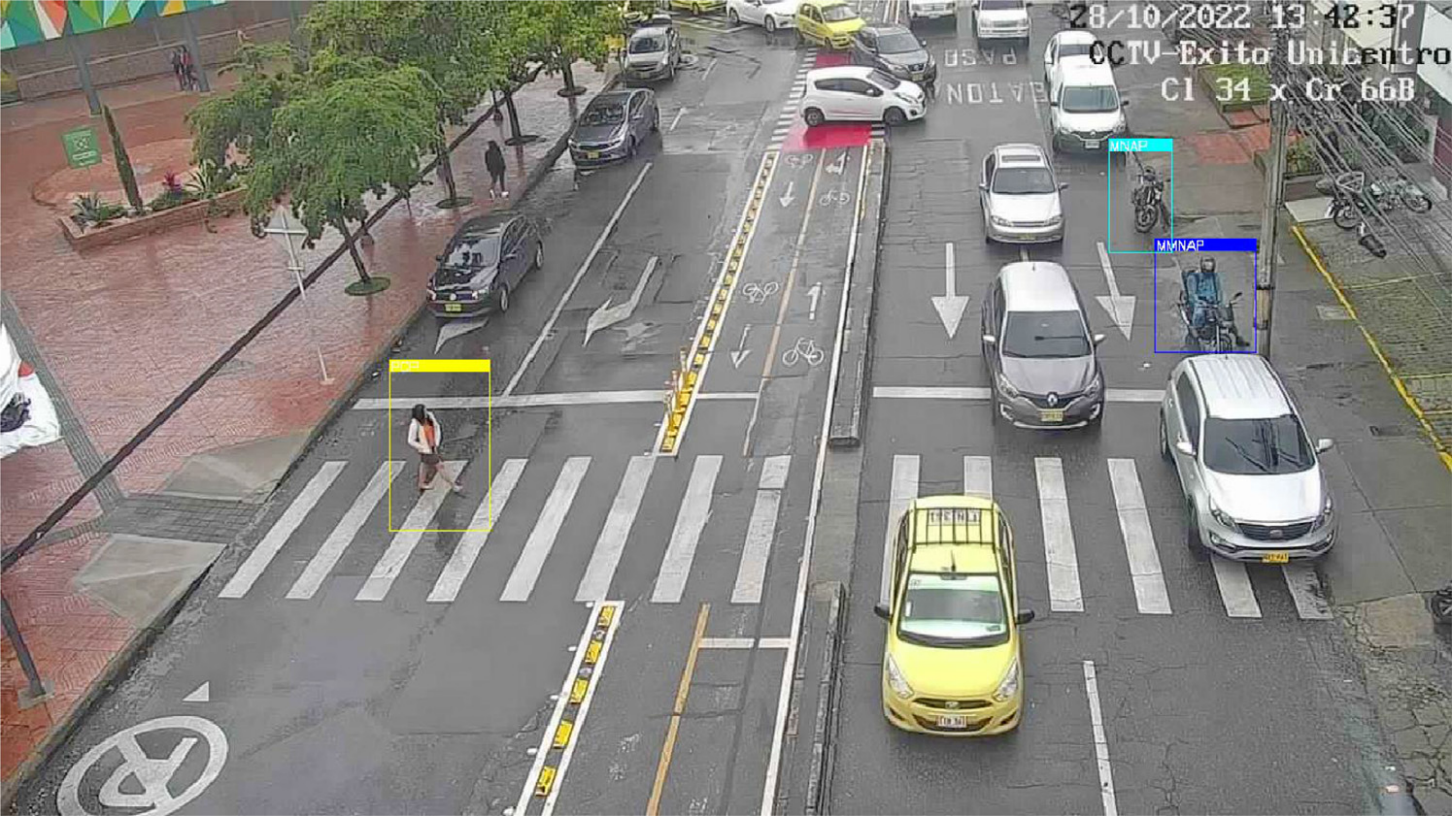
General operation of the label verification system.

Fig. 7 shows that the labeling follows the different classes established for the nth image observed by the nth camera.

So, now, the system allows to record and shows the labels according to the nth image. However, if we review the data structure obtained at the end of the second algorithm, this is equal to the one presented in Fig. 3. Therefore, is necessary to codify an algorithm that makes the conversion between the pickle format constructed and the YOLO txt format wished.

**Fig. 8:**
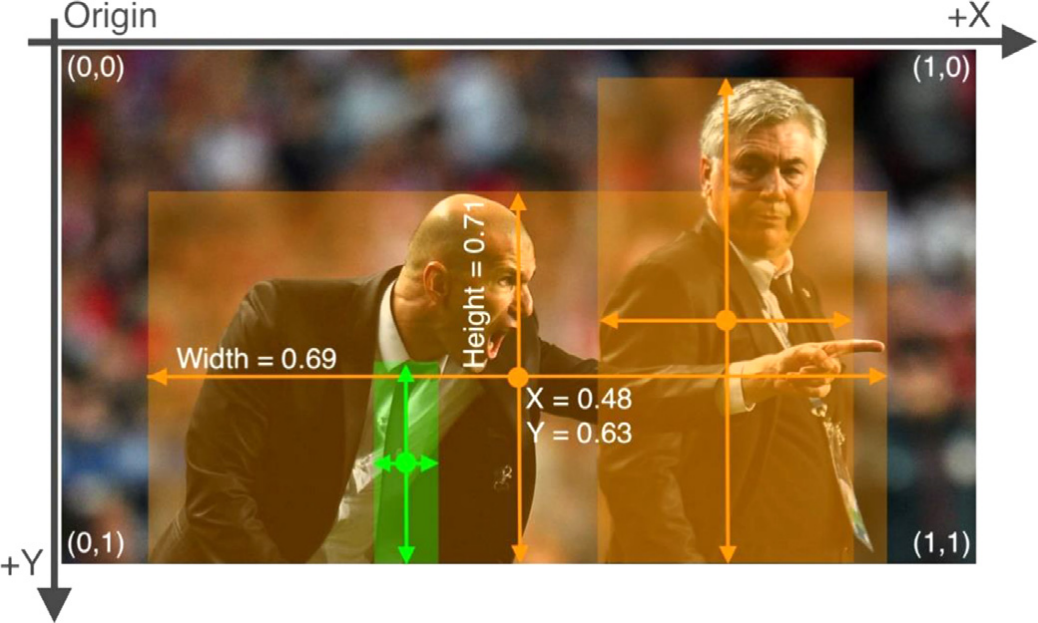
Example YOLO format in function to the boundary box [8].

We resolved the last task by adapting the pickle format designed with the YOLO standard format; according to the conversion is just required to apply the next concept [8],

Fig. 8 illustrates the standard form of a conventional boundary box of the form,

$$X_{min},\;Y_{min},\;X_{max},\;Y_{max},$$

whereX is the width and Y is the height of the nth object's bounding box in the image. This type of boundary box is one count. Therefore, it is necessary to reproduce the interaction shown in the picture to transform any boundary box to its respective YOLO format. The following algorithm facility the interpretation (Algorithm 4).

**Algorithm 4:** Algorithm to make the conversion between pickle format design and YOLO.

Finally, using the last algorithm design, the Dataset available in [1] can be reproduced in raw and labeled images.

## Limitations

Not applicable.

## Ethics statement

The authors have read and followed the *ethical requirements* for publication in Data in Brief and confirm that the current work does not involve human subjects, animal experiments, or any data collected from social media platforms.

## CRediT authorship contribution statement

**Nicolás Hernández Díaz:** Conceptualization, Methodology, Software, Validation. **Yersica C. Peñaloza:** Data curation, Writing – original draft. **Y. Yuliana Rios:** Visualization, Investigation. **Juan Carlos Martinez-Santos:** Supervision. **Edwin Puertas:** Writing – review & editing.

## Data Availability

Dataset for Detecting Motorcyclists in Pedestrian Areas (Original data) (Zenodo).
